# Influence of ankle fracture surgery on glycemic control in patients with diabetes

**DOI:** 10.1186/s12891-016-0987-x

**Published:** 2016-03-23

**Authors:** Seung Yeol Lee, Moon Seok Park, Soon-Sun Kwon, Ki Hyuk Sung, Hyun Soo Jung, Kyoung Min Lee

**Affiliations:** Department of Orthopaedic Surgery, Ewha Womans University Mokdong Hospital, Seoul, Republic of Korea; Department of Orthopaedic Surgery, Seoul National University Bundang Hospital, Seongnam, Republic of Korea; Department of Mathematics, College of Natural Science, Ajou University, Suwon, Republic of Korea

**Keywords:** Ankle fracture, Diabetes, Trauma-induced hyperglycemia, Linear mixed model

## Abstract

**Background:**

Although ankle fracture surgery can affect glycemic control by either trauma-induced stress or a postoperative decrease in physical activity, there is little evidence on this issue. This study aimed to evaluate the influence of ankle fracture surgery on glycemic control and to assess the risk factors for poor glycemic control after surgery in patients with diabetes.

**Methods:**

We reviewed the medical records of consecutive patients with diabetes who underwent open reduction and internal fixation for the treatment of ankle fracture at our hospital. Patients who underwent blood testing, including fasting blood glucose (FBG), glycated hemoglobin (HbA1c), and cholesterol levels, as part of a routine check-up before surgery and again more than 2 times after surgery were included. Changes in blood test results were adjusted by multiple factors using a linear mixed model with sex, age at time of surgery, body mass index (BMI), and type of ankle fracture as the fixed effects and each subject and timing of blood test as the random effects.

**Results:**

Sixty patients were ultimately included in this study. At 1 month postoperatively, mean FBG and cholesterol levels had increased significantly compared with preoperative levels (*p* = 0.011 and 0.024, respectively). After surgery, FBG levels showed an estimated monthly decrease of 2.2 mg/dL (*p* = 0.017). Sex, age at time of surgery, and type of ankle fracture did not significantly affect the monthly change in FBG level. FBG returned to the preoperative level at an estimated period of 8.1 months. BMI significantly affected preoperative FBG level (*p* = 0.015) but not the postoperative change in FBG level (*p* = 0.500).

**Conclusion:**

Ankle fracture surgery increased the FBG level at 1 month postoperatively. FBG levels decreased gradually after surgery at an estimated monthly rate of 2.2 mg/dL. Physicians should be aware of the difficulty in postoperative blood glucose control in patients with diabetes, even several months after surgery.

## Background

Diabetes decreases patients’ quality of life [1] and increases medical costs for patients and society [2]. According to statistics from the Centers for Disease Control and Prevention, in 2011, 25.8 million people (8.3 %) were affected by diabetes in the United States [3]. In particular, 26.9 % of all people over the age of 65 years were affected [3]. Unfortunately, the prevalence of diabetes has increased, [4] and the incidence is expected to increase further with the aging society [5].

With the considerable prevalence and increasing incidence of diabetes, orthopedic surgeons inevitably encounter patients with diabetes. Many studies have reported increased surgical complications, including infection, deep vein thrombosis, and pulmonary thromboembolism, and mortality after orthopedic surgery in patients with diabetes [6–8]. Despite the widespread interest in the adverse effects of diabetes on orthopedic surgery, there is little evidence on the reverse relationship, i.e., the effects of orthopedic surgery on diabetes, even though orthopedic surgery can affect patients’ activity level, which could be associated with metabolic state [9].

The incidence of ankle fractures with features of osteoporotic fracture [10] has increased with the aging society [11, 12]. As the prevalence of diabetes increases, the incidence of ankle fracture in patients with diabetes can also be expected to increase. The influence of diabetes on the management of ankle fracture is far-reaching. For example, diabetes is a risk factor for wound complications after ankle fracture surgery [7] and can result in malunion, delayed union, or Charcot arthropathy [6]. Although acute stress associated with trauma can cause hyperglycemia through increased levels of epinephrine, glucagon, and cortisol, little is known about the effects of ankle fracture surgery on glycemic control [13]. Furthermore, surgery and perioperative immobilization are considered potential reasons that ankle fracture surgery could adversely affect glycemic control in patients with diabetes.

Therefore, we performed this study to evaluate the influence of ankle fracture surgery on glycemic control and to assess the risk factors for poor glycemic control after surgery in patients with diabetes.

## Methods

### Patients and data collection

We reviewed the medical records of consecutive patients with diabetes who underwent open reduction and internal fixation for the treatment of ankle fracture at our hospital between May 2003 and November 2013. Inclusion criteria were patients who underwent blood testing, including assessment of fasting blood glucose (FBG), glycated hemoglobin (HbA1c), and cholesterol levels, as part of a regular check-up before surgery and again more than 2 times after ankle fracture surgery. Exclusion criteria included concomitant injury, pilon fracture, inability to ambulate before or after surgery, and postoperative complications requiring repeat surgery or prolonged immobilization. Age at the time of surgery, sex, body mass index (BMI), and type of ankle fracture were obtained by medical record review. The type of ankle fracture was divided into 2 categories: single (medial or lateral malleolar) and multiple (bi- or trimalleolar). All venous blood sampling was performed in a fasting state in the morning according to our protocol for regular assessment of diabetes. Blood test results were divided into pre- and postoperative and were grouped by month.

### Operative procedure and postoperative protocol

Surgery was performed using spinal or general anesthesia, according to the anesthesiologists’ decision, when swelling subsided and the wrinkle sign appeared. The surgical procedure consisted of open reduction and internal fixation using a plate and screws for the lateral malleolus and a cannulated screw or tension band wiring for the medial malleolus, using separate skin incisions. Fixation of the posterior malleolus was performed percutaneously using a cannulated screw anteroposteriorly when bone fracture segments were displaced more than 2 mm or if they comprised more than one-third of the joint surface on lateral radiography. Syndesmotic fixation was performed in cases of obvious widening of the tibiofibular clear space on stress testing, after fixation of the medial and lateral malleoli. Surgical procedures were performed with a pneumatic tourniquet inflated, followed by compressive dressing with a short-leg-splint immobilization after layer-by-layer wound closure.

Non-weight-bearing ambulation was allowed starting 1 day postoperatively, as tolerated, using a crutch or wheelchair. When the surgical wound was stabilized, a short-leg cast was applied for 2 to 3 weeks postoperatively. Then, a removable short-leg splint was worn, and gentle passive range of motion exercise of the ankle was initiated. At 4 weeks postoperatively, partial weight-bearing walking was allowed using a crutch or walker. Full-weight-bearing walking was initiated at 6 weeks postoperatively, as tolerated, after bone union was confirmed on ankle radiography. Sports activity was allowed at 3 months postoperatively.

### Creation of a linear mixed model

A linear mixed model (LMM) is a parametric linear model for assessing longitudinal data and quantifies the relationships between a continuous dependent variable and various predictor variables; the LMM provides a simple and effective manner to incorporate within- and between-subject variations and the correlation structure of longitudinal data [14]. For each of three indices (FBG, HbA1c, and cholesterol levels), the rate of monthly change was adjusted by multiple factors using an LMM with sex, age at time of trauma, BMI, and type of ankle fracture as the fixed effects and each subject and timing of blood test as the random effects. The covariance structure was assumed as the variance component. The restricted maximum likelihood estimation method was used to produce unbiased estimators. Pre- and postoperative changes in the indices were analyzed separately. Examination of the individual pattern of change along with follow-up time suggested a model with a random slope and random intercept. The models were compared using the Akaike information criterion (AIC) and Bayesian information criterion (BIC). A smaller AIC or BIC value was preferred in terms of model selection. All models had low AIC/BIC scores; therefore, the models were accepted as valid for the estimation of the monthly change in blood indices for diabetes.

### Statistical methods

Descriptive statistics were used to summarize patient demographics and blood test results. To adjust variation of the timing of the test, mean value of each blood index for the first month following surgery was calculated. A paired *t* test was used to analyze the influence of surgery on the mean blood indices measured preoperatively and over the first month following surgery. An LMM was used to model the change rates, assess the covariate effects, and examine the factors contributing significantly to the rate of restoration.

Statistical analyses were conducted using R version 2.15.2 (R Foundation for Statistical Computing, Vienna, Austria, ISBN 3-900051-07-0, URL http://www.r-project.org) and an NLME package. All statistics were 2-tailed, and a *p* value < 0.05 was considered significant.

## Results

Sixty-four patients met the inclusion criteria. After implementation of the exclusion criteria, 60 patients were ultimately included in this study. The mean age at the time of surgery was 63.3 ± 9.8 years (range, 40.2 to 88.7 years), and the mean BMI was 29.5 ± 11.6 kg/m^2^ (range, 14 to 83 kg/m^2^). Twenty-two patients (36.6 %) had a single fracture (medial or lateral malleolar), whereas 38 (63.4 %) had multiple fracture (bi- or trimalleolar; Table 1). The mean number of preoperative blood tests prior to ankle fracture surgery was 8.6 ± 8.8 (range, 2 to 48). Surgeries were performed at 5.3 ± 3.1 days (range, 0 to 14 days) after trauma.

**Table 1:** Patient demographics

Parameter		Value
No. of subjects (male/female)		60 (29/31)
	OP at 2003 to 2008	21
	OP at 2009 to 2013	39
Age at time of surgery (year)		63.3 ± 9.8
BMI (kg/m^2^)		29.5 ± 11.6
Type of ankle fracture	Single	22
	Multiple	38
	FBG	6.9 ± 22.5
First test after surgery^*^	HbA1c	41.3 ± 78.8
	Cholesterol	4.4 ± 18.5
Follow-up duration of each test (day)		
Preoperative follow-up	FBG	135.9 ± 108.4
	HbA1C	164.3 ± 106.1
	Cholesterol	135.6 ± 107.0
	FBG	91.2 ± 114.9
Postoperative follow-up	HbA1c	138.6 ± 120.6
	Cholesterol	87.2 ± 111.2

*No* number, *OP* operation, *BMI* Body Mass Index

^*^Number of days after surgery when the first test was performed

There were no significant changes in FBG (*p* = 0.722), HbA1c (*p* = 0.500), or cholesterol (*p* = 0.166) measurements over the preoperative measurement period. BMI significantly affected the preoperative FBG level, and the FBG level was increased by 2.4 mg/dL for each unit of increase in the BMI (*p* = 0.015). Age and sex did not affect the preoperative FBG level, and HbA1c and cholesterol levels were not affected by age, sex, or BMI.

**Fig. 1:**
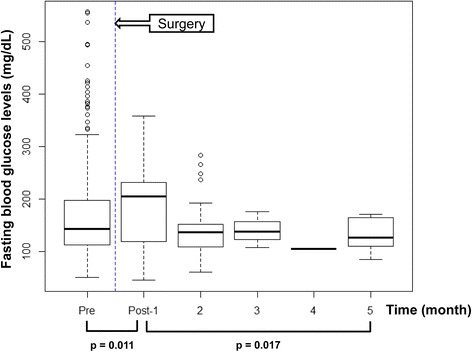
Distribution of fasting blood glucose levels before and after trauma

During the first month following surgery, the mean FBG and cholesterol levels were increased significantly compared with preoperative levels (*p* = 0.011 and 0.024, respectively; Table 2). After surgery, FBG levels showed an estimated monthly decrease of 2.2 mg/dL (*p* = 0.017; Fig. 1). Sex, age at time of surgery, type of ankle fracture, and BMI did not significantly affect the monthly change in FBG levels (Table 3). When patients were divided into two groups according to year of surgery, either 2003 – 2008 or 2009 – 2013, to assess changes in the treatment and control of diabetes, the year of surgery did not affect the monthly change in FBG (estimate = -15.3, *p* = 0.265). FBG returned to the preoperative level at an estimated period of 8.1 months. The mean cholesterol level decreased significantly at 2 months postoperatively (165.6 mg/dL, *p* = 0.027) but showed no significant change at 3 months postoperatively (*p* = 0.535).

**Table 2:** Immediate postoperative changes in fasting blood glucose (FBG), HbA1c, and cholesterol levels

Parameter	Reference range	Preoperative level	1-month postoperative level	*p* value
FBG (mg/dL)	70–110	166.0 ± 81.8	195.1 ± 98.9	0.011
HbA1c (%)	4.0–6.0	7.66 ± 1.42	7.12 ± 0.88	0.404
Clolesterol (mg/dL)	0–200	163.6 ± 41.3	192.8 ± 41.5	0.024

Data are expressed as means

*FBG* fasting blood glucose, *HbA1c* glycated hemoglobin

**Table 3:** Postoperative changes in fasting blood glucose (FBG), HbA1c, and cholesterol levels

	FBG (mg/dL)		HbA1c (%)		Cholesterol (mg/dL)	
	Estimate	*p* value	Estimate	*p* value	Estimate	*p* value
Intercept	183.9	0.001	9.71	<0.001	152.2	<0.001
Gender	6,7	0.632	-0.43	0.178	10.0	0.001
Age at trauma	-0.2	0.749	-0.03	0.083	0.3	0.561
Type of ankle fracture	19.7	0.190	0.01	0.965	10.5	0.649
BMI	-0.4	0.500	-0.02	0.196	0.4	0.380
Time (month)	-2.2	0.039	0.00	0.939	0.8	0.535

*Abbreviations*: *BMI* body mass index, *HbA1c* glycated hemoglobin

HbA1c level was not influenced by surgery (*p* = 0.404) and showed no significant change after ankle fracture surgery (*p* = 0.939).

## Discussion

Ankle fracture surgery can affect glycemic control by either an increase in stress hormones because of trauma or a postoperative change in activity level; however, there is little evidence regarding this issue. Many previous studies have focused on the adverse effects of diabetes on the occurrence of perioperative complications in orthopedic surgery. This study, however, aimed to evaluate the influence of ankle fracture surgery on glycemic control and to assess the risk factors for poor glycemic control after surgery in patients with diabetes. To our knowledge, this is the first study to evaluate hyperglycemia due to ankle fracture surgery in patients with diabetes. Mean FBG and cholesterol levels were found to be significantly increased at 1 month postoperatively, and FBG levels decreased at an estimated rate of 2.2 mg/dL per month postoperatively. A higher BMI increased preoperative FBG level but did not affect postoperative change in FBG level.

Some limitations of this study should be addressed before discussing the study findings in detail. First, this study was retrospective in nature. Data were collected by medical record review and were not strictly controlled. In addition, there was no control group, because the ankle fracture patients without diabetes did not undergo regular check-ups for fasting blood glucose levels. Second, our institution is a tertiary referral center for foot and ankle diseases. The patients who are referred from local clinics to our hospital due to uncontrolled blood glucose levels might be included in this study. Therefore, the study results cannot be applied to the general population. Third, several patients were regularly followed for diabetes at a community hospital; these patients were excluded from the study. Therefore, there might be a selection bias due to including only those who were followed for diabetes at our hospital. Fourth, data on patients’ activity level and daily calorie consumption were not included in detail in this study. Therefore, the exact effects of immobilization on glucose metabolism could not be accurately evaluated. Fifth, types of medications and changes in medications for each subject during follow-up were not considered as an influencing factor on postoperative glycemic control. Changes in diabetic medications could affect the blood glucose levels during the follow-up period. However, medication data could not be included as a potential influencing factor because of the diverse medications of each subject and the small number of subgroups. Therefore, we believe future studies, including a well-controlled prospective study, will be required to evaluate the effect of individual medications on postoperative blood glucose levels.

Previous studies have indicated that obesity is associated with hyperglycemia and an elevated risk for diabetes [4, 15, 16]. The current study showed that BMI was correlated with the preoperative FBG level. Although the data used in this study were unbalanced because the diabetes medication varied for each patient, we used an LMM as an analyzing tool and considered each subject as a random effect to control the data characteristics. Obesity is reported to be responsible for microvascular dysfunction, which results in metabolic insulin resistance [17]. One mechanism linking obesity to microvascular dysfunction is a change in the secretion of adipokines, leading to increased levels of free fatty acids and inflammatory mediators and a decreased level of adiponectin [18]. However, according to our results, BMI did not significantly affect the monthly change in FBG levels postoperatively. This finding suggests that obesity did not affect the acute change in FBG level during surgery or stress. Future studies should investigate the acute response of FBG levels to surgery according to BMI in patients with diabetes.

Increased FBG and cholesterol levels were observed at 1 month after ankle fracture surgery. Hyperglycemia can be a metabolic response to acute stress caused by trauma and subsequent surgery. Trauma-induced hyperglycemia based on the severity of trauma is observed even in patients without diabetes [19]. We believe that stress from surgery itself can affect glucose metabolism, but this issue should be investigated by including patients with conservatively treated ankle fractures. It is interesting that an estimated period of 8.1 months was required for FBG to return to the preoperative level. In addition to trauma-induced hyperglycemia, decreased daily physical activity due to perioperative immobilization is presumed to affect FBG levels. Regular physical activity is essential for glucose homeostasis, [9] and several studies have suggested a beneficial effect of physical activity on hyperglycemia [20, 21].

The HbA1c level was not influenced by ankle fracture surgery, whereas FBG levels increased postoperatively. In diverse international populations, the HbA1c reflects an estimate of the average FBG level during the preceding 8 to 12 weeks [22]. This finding suggests that ankle fracture surgery does not cause long-term changes in FBG levels but does cause an acute increase in FBG levels. To assess the clinical implications of our results, further long-term follow-up studies are required. Recently, several studies have reported that excessive amounts of glucose might disrupt chondrocyte homeostasis via multiple direct and indirect mechanisms and can result in osteoarthritis [23–25]. Exposure to high glucose concentrations favors the chondrocyte catabolic gene expression program, and this mechanism may promote articular cartilage degradation [25]. Osteoarthritic chondrocytes may also be unable to adjust to high extracellular glucose, leading to the intracellular accumulation of glucose [26]. Several previous studies also reported that a hyperglycemia-induced inflammatory response might be one of causes of osteoarthritis [27, 28]. However, these studies were performed in-vitro, [23–26, 28] and in-vivo using a mouse model [27]. There is a lack of evidence regarding the effects of hyperglycemia on cartilage in human models. Further study is required to assess the effects of hyperglycemia postoperatively on chondrocyte homeostasis or the occurrence of postoperative osteoarthritis.

## Conclusions

Ankle fracture surgery increased the FBG and cholesterol levels at 1 month after surgery, and FBG levels decreased at an estimated rate of 2.2 mg/dL per month postoperatively. Sex, age at time of surgery, type of ankle fracture, and BMI did not affect the postoperative change in FBG levels. Finally, FBG returned to the preoperative level at an estimated period of 8.1 months. Physicians should be aware of the difficulty with postoperative blood glucose control in patients with diabetes, even several months after surgery. In addition, physicians should encourage patients to return to daily activity and exercise after cast removal and rehabilitation for effective diabetes management.

### Ethic approval and consent to participate

This retrospective study was approved by the Institutional Review Board of our hospital, a tertiary referral center for foot and ankle diseases. Informed consent was waived because of the retrospective nature of this study.

### Consent for publication

Not applicable.

### Availability of data and materials

The data set supporting the conclusion of this article is available on request to the corresponding author.

## Abbreviations

AIC: Akaike information criterion
BIC: Bayesian information criterion
BMI: body mass index
FBG: fasting blood glucose
HbA1c: glycated hemoglobin
LMM: linear mixed model
